# The potential of microbiota information to better predict efficiency traits in growing pigs fed a conventional and a high-fiber diet

**DOI:** 10.1186/s12711-023-00865-4

**Published:** 2024-01-19

**Authors:** Vanille Déru, Francesco Tiezzi, Céline Carillier-Jacquin, Benoit Blanchet, Laurent Cauquil, Olivier Zemb, Alban Bouquet, Christian Maltecca, Hélène Gilbert

**Affiliations:** 1 GenPhySE, INRAE, ENVT, Université de Toulouse, Castanet-Tolosan, France; 2 France Génétique Porc, 35651 Le Rheu Cedex, France; 3 Department of Agriculture, Food, Environment and Forestry, University of Florence, 50144 Florence, Italy; 4 UE3P, INRAE, Domaine de la Prise, 35590 Saint-Gilles, France; 5 IFIP-Institut du Porc, 35651 Le Rheu Cedex, France; 6 Department of Animal Science, North Carolina State University, Raleigh, NC USA

## Abstract

**Background:**

Improving pigs’ ability to digest diets with an increased dietary fiber content is a lever to improve feed efficiency and limit feed costs in pig production. The aim of this study was to determine whether information on the gut microbiota and host genetics can contribute to predict digestive efficiency (DE, i.e. digestibility coefficients of energy, organic matter, and nitrogen), feed efficiency (FE, i.e. feed conversion ratio and residual feed intake), average daily gain, and daily feed intake phenotypes. Data were available for 1082 pigs fed a conventional or high-fiber diet. Fecal samples were collected at 16 weeks, and DE was estimated using near‑infrared spectrometry. A cross-validation approach was used to predict traits within the same diet, for the opposite diet, and for a combination of both diets, by implementing three models, i.e. with only genomic (Gen), only microbiota (Micro), and both genomic and microbiota information (Micro+Gen). The predictive ability with and without sharing common sires and breeding environment was also evaluated. Prediction accuracy of the phenotypes was calculated as the correlation between model prediction and phenotype adjusted for fixed effects.

**Results:**

Prediction accuracies of the three models were low to moderate (< 0.47) for growth and FE traits and not significantly different between models. In contrast, for DE traits, prediction accuracies of model Gen were low (< 0.30) and those of models Micro and Micro+Gen were moderate to high (> 0.52). Prediction accuracies were not affected by the stratification of diets in the reference and validation sets and were in the same order of magnitude within the same diet, for the opposite diet, and for the combination of both diets. Prediction accuracies of the three models were significantly higher when pigs in the reference and validation populations shared common sires and breeding environment than when they did not (*P* < 0.001).

**Conclusions:**

The microbiota is a relevant source of information to predict DE regardless of the diet, but not to predict growth and FE traits for which prediction accuracies were similar to those obtained with genomic information only. Further analyses on larger datasets and more diverse diets should be carried out to complement and consolidate these results.

**Supplementary Information:**

The online version contains supplementary material available at 10.1186/s12711-023-00865-4.

## Background

Currently, feed costs in pig production represent between 60 and 70% of the total cost of pork production [1]. Feeding pigs with by-products from the agri-food and biofuel industry is an option to reduce feed costs and feed-food competition. In this context, feed efficiency (FE) remains the primary objective of selection. However, recent research has suggested that digestive efficiency (DE), which is the proportion of ingested feed absorbed by the digestive tract, could further improve FE, especially with alternative feedstuff [2]. DE is influenced by the host genetics [2], and has significant genetic correlations with some fecal microbiota traits [3]. Furthermore, fecal microbiota can explain more than 44% of the phenotypic variation for DE traits in pigs [4, 5], and around 20% for FE traits [5–7]. Several studies have investigated the impact of adding microbiota information in models to predict FE and growth phenotypes in pigs. For example, when microbiota information was added to the model to predict average daily gain (ADG), feed conversion ratio (FCR), and daily feed intake (DFI) adjusted for fixed effects, Camarinha-Silva et al. [6] reported low to moderate prediction accuracies (between 0.33 and 0.41) and Aliakbari et al. [7] reported moderate to high prediction accuracies (between 0.56 and 0.64). For residual feed intake (RFI), prediction accuracies were also moderate, around 0.55 [7]. In the study by Camarinha-Silva et al. [6], prediction accuracies for FE and growth traits were higher when microbiota information was added than when host genetics information was added in the model. Similar findings have been demonstrated in rabbits, with an increase from 20 to 46% in prediction accuracy of phenotypes adjusted for fixed effects when microbiota information was added into mixed models [8]. For DE traits in pigs, only one study has reported moderate to high prediction accuracies for organic matter (0.43), dry matter (0.42), and crude protein (0.63) when microbiota information was added to the model [4]. Thus, adding microbiota information could be promising for predicting FE traits in pigs. However, to the best of our knowledge, no study has compared the prediction accuracies of DE traits obtained when microbiota and host genetics information are included in the model. Furthermore, the impact of the diet on prediction accuracies for FE and DE traits has not been reported in the literature. As the composition of the gut microbiota is influenced by the diet and the environment [9–12], it is relevant to disentangle the effects of both factors on prediction accuracy for FE and DE traits.

The goals of the present study were: (1) to evaluate and compare the ability of adding gut microbiota composition and host genetics information to predict DE, FE, and growth traits, and also the impact of including or not gut microbiota composition in the model on the accuracy of estimated breeding values (EBV), and (2) to evaluate the impact on prediction accuracies of training the models on different diets and breeding environments.

## Methods

The study was conducted in accordance with the French legislation on animal experimentation and ethics. The certificate of Authorization to Experiment on Living Animals was issued by the Ministry of Higher Education, Research and Innovation to conduct this experiment under reference number 2017011010237883 at INRAE UE3P—France Génétique Porc phenotyping station (UE3P, INRAE, 2018. Unité expérimentale Physiologie et Phénotypage des Porcs, France, 10.15454/1.5573932732039927E12).

### Experimental design

In this study, we used the same data as in Déru et al. [13]. In total, 1942 purebred Large White (LW) male pigs were reared in 35 consecutive batches between 2017 and 2018 at the INRAE UE3P France Génétique Porc phenotyping station under two dietary conditions. The study was designed to maximize the genetic relationship between the datasets obtained with the two diets: in each pair of full sibs of homogeneous weights, one was fed with a conventional (CO) diet and the other with a high-fiber (HF) diet. All pigs were obtained from 171 sires that were representative of those used in the French Large White collective breeding scheme, and each pair of full-sibs came from a different dam.

Housing conditions and management of pigs are described in detail in Déru et al. [13]. Briefly, upon arrival, pairs of full sibs were separated and allotted in pens of 14 animals. Pigs were raised in post-weaning facilities until 9 weeks of age and fed with a standard two-phase post-weaning dietary sequence. Then, they were moved to the growing-finishing pens without mixing until they reached slaughter weight (i.e. a body weight of 115 kg). For each pair of full sibs, one of the siblings was fed the conventional (CO) diet and the other one was fed a high-fiber (HF) diet. The detailed diet composition is presented in the next section. Each growing-finishing pen contained a single-place electronic feeder equipped with a weighing scale (Genstar, Skiold Acemo, Pontivy, France) to record feed intake and individual body weight of an animal at each visit to the feeder. At a body weight of 115 kg, pigs were fasted for 24 h and then transported to the slaughterhouse. Animals were slaughtered in 89 slaughter batches of approximately 19 pigs each.

### Diets

During the growing-finishing phase, the two sets of pigs were fed two different two-phase rations. First, a growing diet was distributed, then a 5-day transition was organized at 16 weeks of age, and a finishing diet was provided until the end of the test. The HF diet included both insoluble and soluble dietary fibers. The detailed composition of the CO and HF diets is described in Déru et al. [13]. Based on feed formulation, the diets differed in net energy (NE), with 9.6 MJ/kg for the CO diet and 8.2 MJ/kg for the HF diet in both phases (growing and finishing). The diets also differed in neutral detergent fiber (NDF), which was between 13.90 and 15.12% for the CO diet and between 23.82 and 24.46% for the HF diet in the growing and finishing phases. The digestible lysine/NE ratio was identical in both diets, with 0.97–0.99 g/MJ NE in the growing phase and 0.83 g/MJ NE in the finishing phase.

### Recorded traits and sampling

Average daily gain (ADG) and DFI were measured for each animal between 35 and 115 kg body weight. ADG was computed as the ratio between body weight gain and number of days on test. Two FE traits were measured: FCR and RFI. FCR was calculated as the ratio between DFI and ADG and was expressed in kg/kg. RFI, which is the difference between the recorded average daily feed intake and the average daily feed intake predicted for maintenance and production requirements, was determined for the two diets by a single multivariate linear regression using the R software [14] of DFI on ADG, lean meat percentage and carcass yield recorded at slaughter, and average metabolic body weight as described in Déru et al. [13]. Pigs that experienced health problems or injury during the test period were in equal proportion in the two diet groups and were discarded from the analysis. Of the 1942 pigs included in the experiment, 1663 had data available for FCR, ADG, and DFI. Data for RFI were available for 1595 pigs.

DE and fecal microbiota composition were also determined for the animals included in this study. A unique fecal sample was collected at 16 weeks of age, just before the feed transition between the growing and finishing phases, for DE determination and microbiota composition analyses. Feces were collected in a piping bag for each pig and manually homogenized. About 50 g of feces were stored in plastic containers at − 20 °C until further analyses to predict DE traits. Samples were freeze-dried and ground with a grinder (Grindomix GM200, Retsch). Then, DE was computed using digestibility coefficients (DC) that represent the proportion of nutrients absorbed by the digestive tract, including energy, nitrogen, and organic matter. DC were predicted using near infrared spectrometry (NIRS) analyses of these samples, as described in detail in Déru et al. [2]. The prediction equations for DC of organic matter, nitrogen, and energy were reliable, with cross-validation R2 values higher than 0.89 [15]. Data for DC were available for 1242 of the pigs included in the experiment, with 654 fed a CO diet and 588 fed an HF diet.

Another fraction of the feces samples was used to assess microbiota composition for each pig, as described in the next section.

### Preparation and sequencing of microbiota DNA

As an approximation for the gut microbiota composition, fecal samples were collected since the compositions of the fecal microbiota and the large intestine are similar [16]. Microbiota composition was analyzed by sequencing the ribosomal 16S gene. The details of the preparation and sequencing of microbiota DNA are in Déru et al. [3], and briefly summarized in the next paragraph. The V3–V4 regions of 16S rRNA were sequenced. Filtering and trimming of the high-quality sequence reads were done using the DADA2 package in the R software [17]. Chimeras were removed, and no further clustering was applied, thus in this study, operational taxonomic units (OTU) were equivalent to amplicon sequence variants. Subsequently, the final OTU abundance table was obtained. This step was followed by taxonomic annotation using the *assignTaxonomy* function of DADA2 with the Silva v132 database [18]. The file obtained was rarefied to 10,000 counts per sample using the phyloseq package [19] and information was available for 1564 pigs. In total, 14,366 OTU were kept in the abundance tables for 812 pigs fed the CO diet and 752 pigs fed the HF diet. Sequence information for the current study was deposited in the Short-Read Archive with accession number PRJNA741111.

### Genotyping

In total, 1691 animals were genotyped with the 70K single nucleotide polymorphism (SNP) GeneSeek GGP Porcine HD chip. Quality control (QC) was carried out using the following criteria: a call rate per individual, i.e., the percentage of genotypes present per individual, higher than 95%, and a SNP call rate, i.e., the percentage of genotypes by SNP, higher than 95%. SNPs with a minor allele frequency lower than 5% or with a significant deviation from the Hardy–Weinberg equilibrium (*P* < 0.000001) and SNPs on sex chromosomes or not mapped were deleted. This QC was performed with the PLINK 1.09 software [20]. After QC, 1687 animals and 48,919 SNPs were available for further analyses.

### Prediction of phenotypes using microbiota and genomic data

Only animals with microbiota, genomic, and phenotypic information for the ADG, DFI, FE, and DE traits were kept for subsequent analyses, which represented 1082 animals in 32 batches, including 566 animals fed the CO diet and 516 animals fed the HF diet.

#### Microbiota and genomic covariance matrices

To account for the microbiota and genetic effects in the linear mixed models, a microbiota covariance matrix $\left( {\mathbf{M}} \right)$ and a genomic relationship matrix $\left( {\mathbf{G}} \right)$ were obtained based on microbiota and SNP data, respectively. The microbiota covariance matrix was obtained following the method proposed by Ross et al. [21] and as described in Déru et al. [5], by retaining the OTU that were present in more than five samples and that had an average abundance higher than 0.001% (2399 OTU). The ${\mathbf{M}}$ matrix was computed for each diet and for the combination of both diets depending on the scenario, and was obtained as follows:

$${\mathbf{M}} = \frac{{{\mathbf{SS}}^{{\text{T}}} }}{{\text{n}}},$$

with matrix ${\mathbf{S}}$ of dimension ${\text{p}} \times {\text{n}}$, where ${\text{p}}$ is the number of animals and ${\text{n}}$ is the number of OTU, constructed from elements ${\text{s}}_{{{\text{jk}}}}$ containing the log-transformed count of OTU ${\text{k}}$ for animal ${\text{j}}$ [21]. Ones were added to all elements to ensure the subsequent log-transformation of null elements. The log-transformed counts of the OTU were then centered and scaled.

The genomic relationship ${\mathbf{G}}$ was computed according to the first method of VanRaden [22] with the AGHmatrix package in R [23].

#### Models

A preliminary analysis in which additive genomic and microbiota effects were ignored was carried out to determine the fixed and random effects to be included in subsequent analyses using a linear mixed model implemented with the “lme4” and “lmerTest” R packages [24, 25]. Only the effects significant at a threshold of 5% were retained.

To predict the growth, FE and DE traits from microbiota information only, genomic information only, and from the combination of both, three Bayesian linear regression models were fitted with the R package BGLR [26] as summarized in this paragraph: model Gen included only a genomic effect, model Micro only a microbiota effect, and model Micro+Gen both effects as follows:

model Gen $${\mathbf{y}} = {\mathbf{X}{\boldsymbol{\upbeta}}} + {\mathbf{Zu}} + {\mathbf{e}},$$

model Micro $${\mathbf{y}} = {\mathbf{X}{\boldsymbol{\upbeta}}} + {\mathbf{Wm}} + {\mathbf{e}},$$

model Micro+Gen ${\mathbf{y}} = {\mathbf{X}{\boldsymbol{\upbeta}}} + {\mathbf{Zu}} + {\mathbf{Wm}} + {\mathbf{e}},$

where ${\mathbf{y}}$ is the vector of phenotypes for a given trait (with length equal to the number of individuals with phenotypes), ${\mathbf{X}}$ is the incidence matrix relating observations to fixed effects, ${{\varvec{\upbeta}}}$ is the vector of fixed effects described in the next section, ${\mathbf{Z}}$ is the incidence matrix for the genetic effect of the individual, and ${\mathbf{u}}\sim {\text{N}}\left( {0,{\mathbf{G}}\sigma_{u}^{2} } \right)$ is the vector of random additive genetic effects for the considered trait (with length equal to the number of individuals in ${\mathbf{G}}$) and $\sigma_{u}^{2}$ is the additive genetic variance. ${\mathbf{W}}$ is the incidence matrix for the microbiota effects, and ${\mathbf{m}}\sim {\text{N}}\left( {{\mathbf{0}},{\mathbf{M}}\sigma_{m}^{2} } \right)$ is the vector of microbiota effects for the considered trait (with length equal to the number of individuals in ${\mathbf{M}}$), and with $\sigma_{m}^{2}$ the microbiota variance. Finally, ${\mathbf{e}}\sim {\text{N}}\left( {{\mathbf{0}},{\mathbf{I}}\sigma_{e}^{2} } \right)$ is the vector of the residual random effect (with length equal to the number of individuals with phenotypes), and with ${\mathbf{I}}$ the identity matrix, and $\sigma_{e}^{2}$ the residual variance. Since all individuals in the model had a record for genomic and microbiota information, ${\mathbf{W}}$ and ${\mathbf{Z}}$ could be substituted with ${\mathbf{I}}$. The Micro+Gen model assumed a null covariance between ${\mathbf{u}}$ and ${\mathbf{m}}$.

For all three models, the residual, microbiota, and genetic variances were assigned scaled-inverse Chi-square densities as prior density, with hyperparameters of 5 degrees of freedom and a scale parameter based on the sample variance of the phenotypes, as proposed by default in the BGLR package [26]. A Gaussian prior with a mean of zero and a variance equal to 10^10^ was assigned to the fixed effects. Further details about the models implemented in BGLR, such as the number of iterations, burn-in, and thinning, are in Déru et al. [5].

### Cross-validation approach

**Fig. 1:**
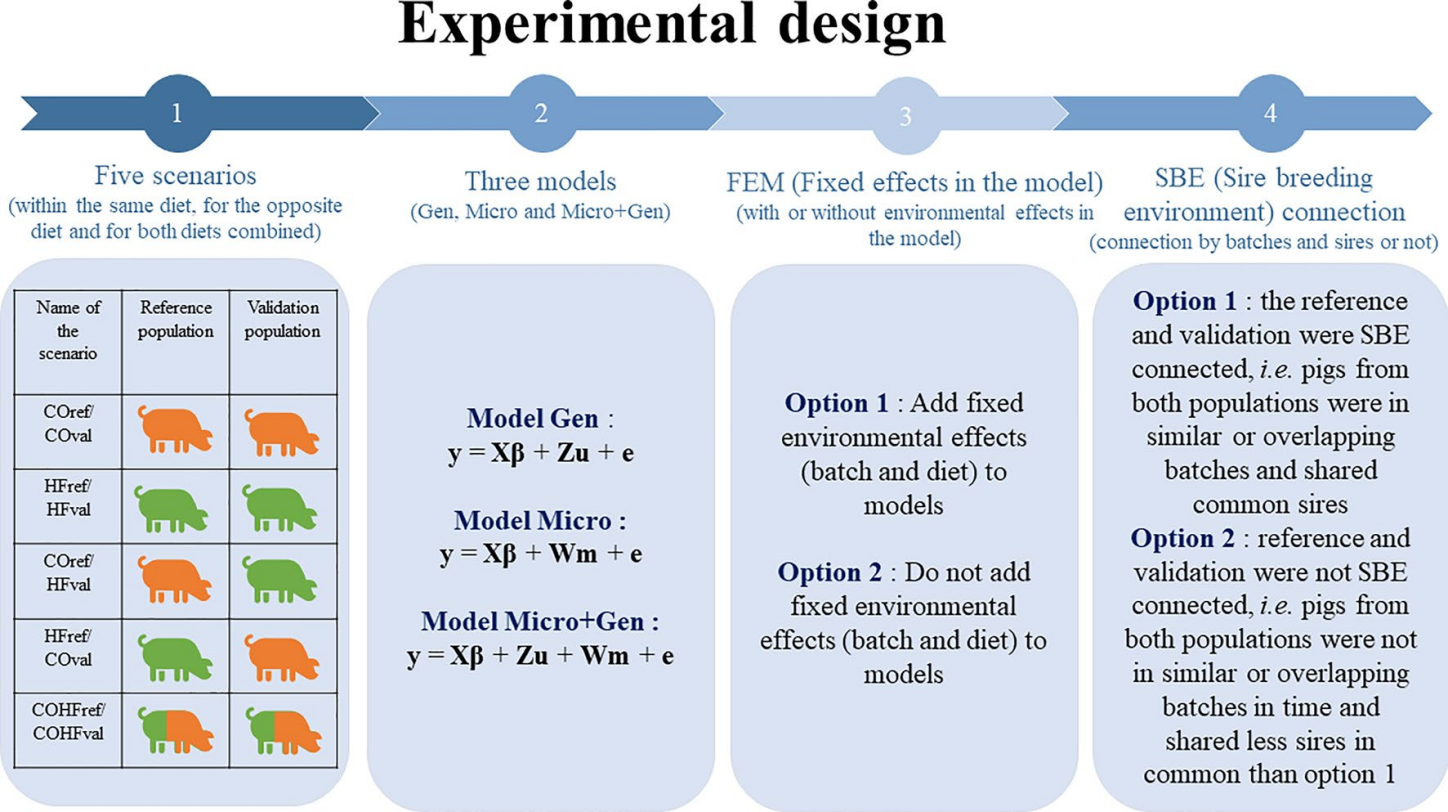
Experimental design for cross-validation study. Pigs in orange = pigs fed a conventional diet; pigs in green = pigs fed a high-fiber diet. ${\mathbf{y}}$ is the vector of phenotypes for a given trait. ${\mathbf{X}}$ is the incidence matrix relating observations to fixed effects. ${{\varvec{\upbeta}}}$ is the vector of fixed effects described in the next section. ${\mathbf{Z}}$ is the incidence matrix for the genetic effect of the individual, and ${\mathbf{u}}\sim {\text{N}}\left( {{\mathbf{0}},{\mathbf{G}}\sigma_{u}^{2} } \right)$ is the random vector of additive genetic effects for the considered trait, with $\sigma_{u}^{2}$ is the additive genetic variance. ${\mathbf{W}}$ is the incidence matrix for the microbiota effects, and ${\mathbf{m}}\sim {\text{N}}\left( {{\mathbf{0}},{\mathbf{M}}\sigma_{m}^{2} } \right)$ is the vector of microbiota effects for the considered trait, with $\sigma_{m}^{2}$ the microbiota variance. Finally, ${\mathbf{e}}\sim {\text{N}}\left( {{\mathbf{0}},{\mathbf{I}}\sigma_{e}^{2} } \right)$ is the vector of residual random effect, with ${\mathbf{I}}$ the identity matrix, and $\sigma_{e}^{2}$ the residual variance

A forward cross-validation approach was used (see Fig. 1).

#### Scenarios

The first objective of the investigation was to test the influence of diet on the prediction accuracy for different phenotypes. Five scenarios were constructed to test prediction accuracy within the same diet, for the opposite diet, and for the combination of both diets, as presented in step 1 of Fig. 1. Within the same diet, the first scenario consisted of pigs fed a CO diet in both the reference and validation populations (COref/COval), and the second scenario consisted of pigs fed an HF diet in both the reference and validation populations (HFref/HFval). To test the prediction accuracy for the opposite diet, two scenarios were constructed with pigs fed the CO and HF diets, respectively, in the reference and validation populations (COref/HFval), and vice-versa (HFref/COval). In addition, a scenario combining both diets in equal proportions in the reference and validation populations (COHFref/COHFval) was constructed. Four folds were taken with different reference and validation populations, with 310 pigs in the reference and 45 pigs in the validation population. However, given the number of pigs available and the different scenarios, the reference and validation populations were not independent of each other; the percentage of shared individuals from one sampling to another ranged from 28 to 92% for the reference and from 0 to 77% for the validation population (see Additional file 1: Fig. S1).

#### Effect of breeding environment on prediction accuracy

**Fig. 2:**
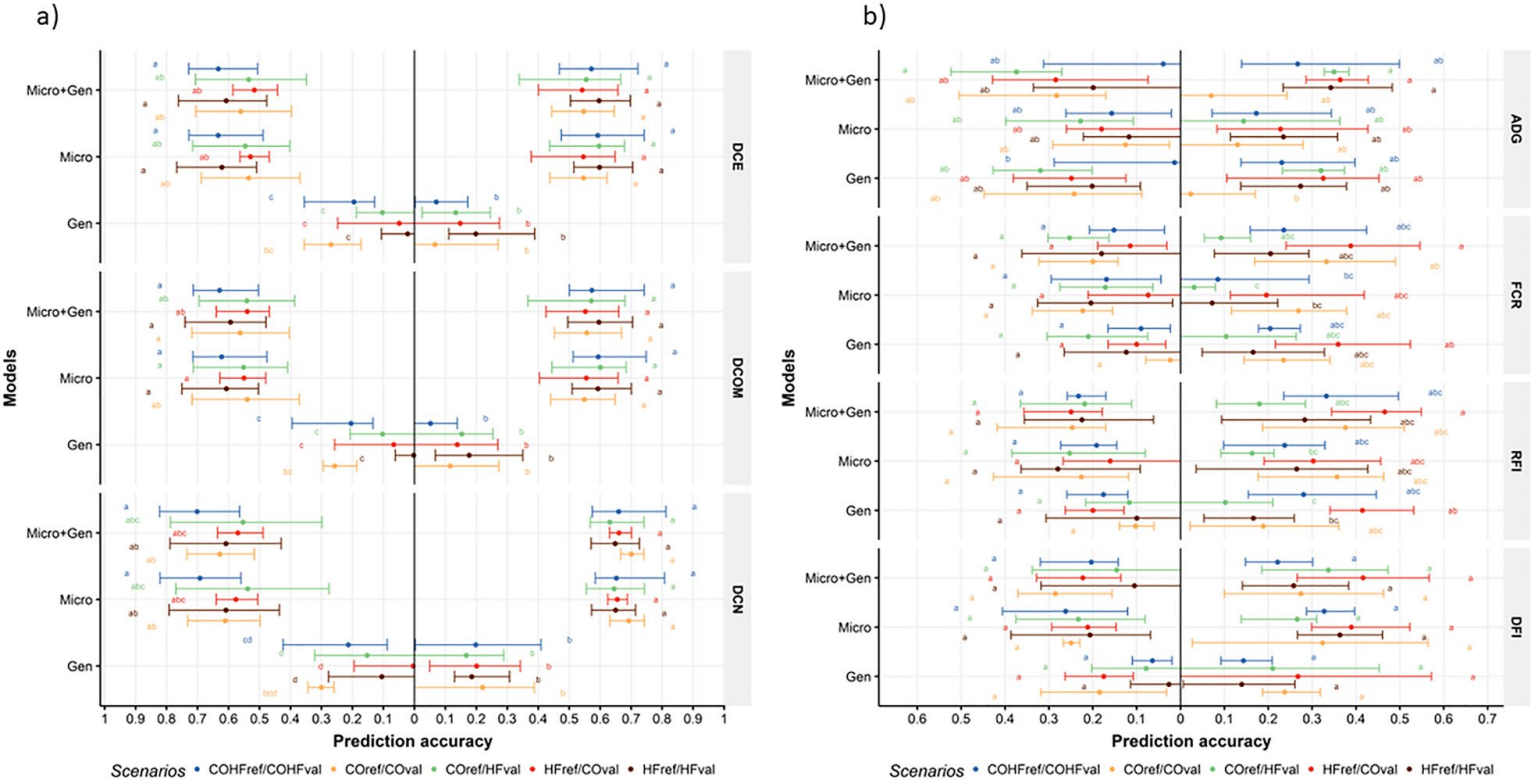
**a** Mean of prediction accuracy for the digestibility coefficient of energy (DCE), organic matter (DCOM) and nitrogen (DCN) phenotypes, depending on the model (Gen, Micro, and Micro+Gen), the scenario (COval/COref, HFval/HFref, COval/HFref, HFval/COref, and COHFval/COHFref) and if reference and validation populations shared (on the right) or did not share (on the left) a common environment, with minimum and maximum values associated. **b** Mean of prediction accuracy for average daily gain (ADG), feed conversion ratio (FCR), residual feed intake (RFI) and daily feed intake (DFI) phenotypes, depending on the model (Gen, Micro, and Micro+Gen), the scenario (COval/COref, HFval/HFref, COval/HFref, HFval/COref, and COHFval/COHFref) and if reference and validation populations shared (on the right) or did not share (on the left) a common environment, with minimum and maximum values associated. Prediction accuracies were compared to each other for the same trait within the reference populations connected by the batches or not. Model Gen: model with only genomic information; Model Micro: model with only microbiota information; Model Micro+Gen: model with microbiota + genomic information combined. Ref. and val. environmentally connected = reference and validation populations environmentally connected; Ref. and val. environmentally not connected = reference and validation populations environmentally not connected. COref/COval: pigs were fed a conventional diet in the reference and the validation populations; HFref/HFval: pigs were fed a high-fiber diet in the reference and the validation populations; COref/HFval: pigs were fed a conventional diet in the reference and a high-fiber diet in the validation population; HFref/COval: pigs were fed a high-fiber diet in the reference and a conventional diet in the validation population; COHFref/COHFval: pigs were fed a conventional + a high-fiber diet in the reference and a conventional + a high-fiber diet in the validation population

To assess the influence of the environment in which the pig was bred, as captured by the diet and batch effects, on the accuracy of predictions, two approaches were evaluated: (1) estimation of the prediction accuracies when the fixed effects of batch and diet were included or not in the model, referred to “FEM” (fixed effects in the model) (Step 3 in Fig. 1), and (2) estimation of the prediction accuracies when the reference and validation populations shared a common paternal family and breeding environment, ‘SBE’ (sire breeding environment) (Step 4 in Fig. 1).

The initial hypothesis was that not correcting for environmental effects would improve the prediction accuracy of the phenotypes, especially in the Micro model in which the gut microbiota composition is sensitive to breeding environmental conditions. For this purpose, prediction accuracies were first calculated only with phenotypes corrected for DFI for DC, for weight at the end of the post-weaning phase for ADG and FCR, and for weight at the end of the test for DFI and RFI. In a second step, prediction accuracies were obtained for phenotypes corrected for all the previous effects and for batch and diet fixed effects.

Then, to evaluate the impact of the breeding environmental connection (by batches) between the reference and the validation populations on prediction accuracy, two different options were considered in each of the above scenarios: the reference and validation populations were environmentally connected, i.e., pigs from both populations were in similar or overlapping batches at a given time, and reference and validation populations were not environmentally connected, i.e., pigs from both populations were not in similar or overlapping batches at a given time. Note that individuals in connected batches also shared more sires than non-connected batches (see Additional file 2: Table S1) because batches were processed in chronological order; pigs that were together in the building simultaneously were more likely to have a common sire than those separated by several months. So, when reference and validation populations were environmentally connected, they were also genetically connected, hence the SBE connection.

#### Prediction accuracy

First, prediction accuracy of traits from microbiota and/or genomic information was estimated as the correlation between the model prediction and the phenotype adjusted for fixed effects. Phenotypes were adjusted for fixed effects (y*) using the Gen, Micro, and Micro+Gen models, respectively, depending on the prediction model and using the whole dataset. The Pearson rank correlations between the y* values obtained from the three models and the full dataset were, in all cases, high, between 0.89 and 0.99. Depending on the model (Gen, Micro, and Micro+Gen), for each sampling, the correlation between the phenotype adjusted for fixed effects y* and the vector of predictions (EBV in model Gen, estimated microbiota value (EMV) in model Micro, and EMV and EBV, both separated and combined in model Micro+Gen,) was calculated. The prediction accuracy (${\text{r}}$) was obtained for each cross-validation run of each model and then averaged for the four samplings within each scenario, model, FEM, and SBE, as shown in Fig. 1.

However, only breeding values are of interest for genetic selection. Thus, the ability to better predict or not EBV when gut microbiota information is included in the model was evaluated. For this purpose, the prediction accuracies between y*, which is the best proxy for the true breeding values in our study, and the EBV were compared between the Gen and the Micro+Gen models.

### Post-hoc analysis

The impact of different effects on prediction accuracy was evaluated by analysis of the variance (ANOVA) that was performed with the Anova() R function [14]. The five effects were (1) the scenario (COref/COval, HFref/HFval, COref/HFval, HFref/COval, and COHFref/COHFval); (2) the model (Gen, Micr, and Micro+Gen); (3) the nature of the trait (growth/feed or digestive efficiency trait); (4) the SBE connection; and (5) the FEM (diet and batch).

Then, multiple comparisons of prediction accuracies were performed. The assumptions of normality and homogeneity of variances were not respected in all cases; thus, multiple comparisons of means were performed with a Welch test. Multiple comparisons were performed with the *pairwise.t.test()* function of the stats package in R, and the options var.eq=FALSE and pool.sd=FALSE. The significance level was corrected with a Bonferroni correction with the option p.adjust.method="bonf". Prediction accuracies were arbitrarily considered low when they were lower than 0.40, moderate when they were between 0.40 and 0.60, and high when they were higher than 0.60.

## Results

### Effect of different factors on prediction accuracy

The impact of the different factors on prediction accuracy is reported in Table 1. All the effects were significant (*P* < 0.001), except for the FEM effect (*P* = 0.37). Prediction accuracies were significantly higher (*P* < 0.001) when pigs of the reference and validation populations were SBE connected (0.27 for growth and FE traits; and 0.47 for DE traits) than when they were not SBE connected (0.16 for growth and FE traits and 0.40 for DE traits). Prediction accuracies for the DE traits were significantly higher than those estimated for growth and FE traits (0.21 vs. 0.43, *P* < 0.001). A significant effect of the scenarios on prediction accuracy was observed (*P* < 0.001). Since prediction accuracy was not affected by the inclusion/exclusion of environmental effects, only the results, including environmental effects in the models, will be presented in the following.

**Table 1:** Influence of five design effects on prediction accuracy: degrees of freedom, F-value, and associated P-value in combined analyses of variance

Effects	Degrees of freedom	*F*-value	*P*-value
Scenario^a^	4	10.07	4.67 × 10^–5^
SBE^b^	1	109.49	< 2.2 × 10^–16^
Type of trait (growth/feed vs. digestive)	1	634.61	< 2.2 × 10^–16^
Model^c^	2	109.49	< 2.2 × 10^–16^
FEM^d^	1	0.81	0.37
Residuals	1670		

^a^Five scenarios were analysed—COref/COval: pigs were fed a conventional diet in the reference and the validation populations; HFref/HFval: pigs were fed a high-fiber diet in the reference and the validation populations; COref/HFval: pigs were fed a conventional diet in the reference population and a high-fiber diet in the validation population; HFref/COval: pigs were fed a high-fiber diet in the reference population and a conventional diet in the validation population; COHFref/COHFval: pigs were fed a conventional + a high-fiber diet in the reference population and a conventional + a high-fiber diet in the validation population

^b^SBE: sire breeding environment; the impact of a common sire breeding environment between the reference and validation populations was tested

^c^Three models were used: Micro (with only microbiota information), Gen (with only genomic information), and Micro+Gen (with both microbiota and genomic information)

^d^FEM: fixed effects in the model, for each trait, two phenotypes were studied: phenotypes corrected or not for environmental effects (diet and batch)

### Phenotypic predictions

The prediction accuracies for each DE trait, scenario, model, and SBE connection are presented in Fig. 2a, and for growth and FE traits in Fig. 2b. The detailed values are in Additional file 2: Table S2.

#### Impact of adding microbiota vs. genomic data on prediction accuracy for feed and digestive efficiency traits

For the three DE traits, prediction accuracies were low in model Gen (between − 0.01 and 0.30), moderate to high in models Micro (between 0.54 and 0.69) and Micro+Gen (between 0.53 and 0.70), i.e., prediction accuracies were not significantly different between the Micro and Micro+Gen models but significantly lower in the Gen model. For the four growth and FE traits, prediction accuracies were low to moderate, from 0.01 to 0.47, and not significantly different between the three models. The prediction accuracies of model Gen were of the same order of magnitude for the DE traits and the growth and FE traits. On the contrary, prediction accuracies were significantly higher in model Micro and Micro+Gen for the DE traits than for the growth and FE traits.

Adding the host genetics information in the model yielded low to moderate prediction accuracies for the FE and DE traits, while adding microbiota information in the model considerably increased the prediction accuracies for the DE traits (> 0.53), but not for the FE traits (< 0.47).

#### Influence of the diet on prediction

In the global model presented in Table 1, the scenario effect significantly impacted prediction accuracies (*P* < 0.0001). In general, across scenarios, models, SBE connection, and traits, prediction accuracies were significantly higher for the COHFref/COHFval (0.36) than for the COref/HFval (0.27) and HFref/HFval (0.27) scenarios. However, when we examined the individual traits, scenarios, models, and SBE connections separately, as shown in Fig. 2a, b, no significant differences were observed between the scenarios, except in the case of RFI with model Gen. In this particular case, when the reference and validation populations were SBE connected, prediction accuracy was notably lower for scenario COref/HFval (0.10) compared to scenario HFref/COval (0.41). Within each model and for each trait, prediction accuracies were not significantly different between the COref/COval and HFref/HFval scenarios. Therefore, there was no difference in prediction accuracy between diets. The across-diet scenarios also showed prediction accuracies of the same order of magnitude than the within-diet scenarios. For RFI, prediction accuracies were higher when pigs were fed an HF diet in the reference population and a CO diet in the validation population (HFref/COval) than for the opposite case (COref/HFval). When both diets were combined together in the reference and validation populations (COHFref/COHFval scenario), the prediction accuracies did not differ from those obtained within and across diets for all traits. Thus, the differences between the scenarios in prediction accuracy were small, which suggests a weak or null influence of the pig diet on the predictability.

#### Influence of the breeding environment on prediction accuracy

Overall, prediction accuracies were significantly higher when pigs of the reference and validation populations were SBE connected than when they were not connected by SBE (*P* < 0.001). With the Gen model and for growth and FE traits, prediction accuracies ranged from 0.02 to 0.41 when reference and validation populations were connected by SBE, and they were significantly higher compared to when reference and validation populations were not connected by SBE (from 0.01 to 0.32) (*P* < 0.001). The same observation was made for the DE traits for which prediction accuracies were significantly higher when pigs of the reference and validation population were connected by SBE (from 0.05 to 0.30) than when they were not (from − 0.01 to 0.30) (*P* < 0.001). With the Micro model, prediction accuracies were also higher when pigs were connected by SBE for growth and FE traits (from 0.03 to 0.36), and DE traits (from 0.55 to 0.69) than when they were not (from 0.07 to 0.28 for growth and FE traits, and from 0.53 to 0.63 for DE traits) (*P* < 0.001). The same observation was made with model Micro+Gen for growth, FE, and DE traits (*P* < 0.001).

#### Contribution of microbiota and genomics to the prediction of the adjusted phenotypes in the Micro+Gen model

The correlations estimated between the phenotypes corrected for fixed effects and EBV and EMV separately and combined in model Micro+Gen are in Table 2 and compared with those obtained in models Micro and Gen.

**Table 2:** Mean of the prediction accuracy of phenotypes in the COHFref/COHFval scenario with common environmental conditions for the reference and validation populations: comparison across three models (Gen, Micro and Micro+Gen), with the estimated microbiota value (EMV) and estimated breeding value (EBV) separately, or combined in the Micro+Gen model

Trait^a^	Model Gen^b^	Model Micro^c^	Model Micro+Gen^d^		
	r(y*, EBV)	r(y*, EMV)	r(y*, EBV)	r(y*, EMV)	r(y*, EMV+EBV)
DCE	0.07 [0.00; 0.17]	0.59 [0.47; 0.74]	0.06 [0.00; 0.10]	0.58 [0.47; 0.73]	0.57 [0.47; 0.72]
DCOM	0.05 [− 0.06; 0.14]	0.59 [0.51; 0.75]	0.05 [0.03; 0.08]	0.58 [0.51; 0.74]	0.57 [0.50; 0.74]
DCN	0.20 [0.00; 0.41]	0.65 [0.58; 0.80]	0.19 [0.09; 0.31]	0.65 [0.59; 0.80]	0.66 [0.57; 0.81]
ADG	0.23 [− 0.14; 0.40]	0.17 [0.07; 0.34]	0.23 [0.14; 0.38]	0.18 [0.05; 0.33]	0.27 [0.14; 0.50]
FCR	0.20 [0.18; 0.27]	0.09 [0.00; 0.29]	0.20 [0.15; 0.23]	0.11 [0.02; 0.34]	0.24 [0.16; 0.42]
RFI	0.21 [0.09; 0.45]	0.24 [0.10; 0.33]	0.29 [0.15; 0.46]	0.24 [0.12; 0.28]	0.33 [0.35; 0.50]
DFI	0.14 [0.09; 0.21]	0.33 [0.29; 0.40]	0.06 [0.14; 0.38]	0.30 [0.26; 0.33]	0.22 [0.15; 0.30]

^a^*DCE* digestibility coefficient of energy, *DCOM* digestibility coefficient of organic matter, *DCN* digestibility coefficient of nitrogen, *ADG* average daily gain, *DFI* daily feed intake, *FCR* feed conversion ratio, *RFI* residual feed intake

^b^Model Gen = model with only genomic information

^c^Model Micro = model with only microbiota information

^d^Model Micro+Gen = model with microbiota + genomic information combined

For DE traits, prediction accuracies estimated in model Micro+Gen ranged from 0.05 to 0.19 when using only the EBV, whereas they ranged from 0.58 to 0.65 when only using the EMV portion. Moreover, these prediction accuracies were identical (within the limit of plus or minus 0.01) to those obtained in the Gen and Micro models, which indicates that, for DE traits, prediction accuracies were driven by the microbiota information when both genomic and microbiota information were included in the model.

For growth and FE traits, prediction accuracies ranged from 0.06 to 0.29 for the Gen model and from 0.11 to 0.30 for the Micro model. Prediction accuracies were equivalently driven by the microbiota and genomics information, and prediction accuracies obtained with the Micro+Gen model were close to those obtained with the Gen and Micro models; thus in our study, when both microbiota and genomics information were included in the model, they contributed equally to prediction accuracy for growth and FE traits.

In addition, based on this analysis, EBV were more accurately predicted when microbiota information was included in the model, as can be seen from the reduced ranges of minimum and maximum values for r(y*, EBV) in model Micro+Gen compared to r(y*, EBV) in model Gen, for all traits except DFI.

## Discussion

In this study, we evaluated the effect of including microbiota information only or with host genomic information, in models to predict DE, growth, and FE traits. In addition, we evaluated the impact of different diets and breeding environments on the prediction accuracy of these traits. Our results support the hypothesis that fecal microbiota is a relevant source of information for predicting DE traits but less so for predicting FE traits. Interactions with diets seemed limited and did not affect prediction accuracies.

### Gut microbiota composition: a relevant source of information to predict digestive efficiency traits

In our design, for growth, FE, and DE traits, including host genetics information only resulted in low to moderate prediction accuracies and for growth and FE traits, including microbiota information only also resulted in low to moderate prediction accuracies and was not a better predictor than host genetics. However, microbiota information was a good predictor for DE traits, with moderate to high prediction accuracies (> 0.53).

#### Limited potential of gut microbiota to improve prediction accuracy of feed efficiency traits

The prediction accuracies obtained for FE traits in our study were comparable to those reported by Camarinha-Silva et al. [6]. Specifically, for ADG, FCR, and DFI, they found prediction accuracies ranging from 0.33 to 0.41 in the Gen model and from 0.20 to 0.35 in the Micro model [6], which is consistent with our results. Similarly, Aliakbari et al. [7] reported non-significant differences in prediction accuracies for FE traits between the models incorporating microbiota information and those including genetic information. However, in their study conducted in 2022, the Micro model showed higher prediction accuracy estimates than those in our study, ranging from 0.61 to 0.81 for FCR, RFI, DFI, and ADG. It is worth noting that Aliakbari et al. [7] also used experimental French Large White pig lines but with a population that was developed over 11 generations of divergent selection for RFI and with a smaller sample size (604 vs. 1082). These discrepancies between studies could account for the differences in prediction accuracy estimates.

Combining genomic and microbiota information in the previous study led to a slight increase in prediction accuracy, ranging from 0.05 to 0.19 points [7]. However, in our study, no significant improvement was observed when both factors were considered together [5]. Although microbiability estimates suggested that the genetic and microbiota effects could be distinguished in our dataset, the lack of improvement in prediction accuracy when the second random effect was included in the model may be attributed to partial confounding effects between the genomic and microbiota effects, as previously suggested in the literature [27–30]. To address the limitations of assuming independence between these effects, advanced models have been proposed to disentangle the two factors and better capture the interaction between genetics and microbiota. Further research in this area is warranted [27, 29, 30].

Several studies have indicated that the contribution of gut microbiota to the variability of FE traits is modest, ranging from low to moderate, with a percentage of variance around 20% [6, 7, 27, 31, 32]. Therefore, the relatively low contribution of the microbiota composition to the prediction of these traits was expected.

Based on our results, microbiota and genomics information provide the same level of phenotypic prediction for FE traits. Based on the EBV, it appears that for these traits, when genetic and microbiota effects are combined in the same model, the prediction accuracies of EBV were higher for ADG, FCR, and RFI. David and Ricard [33] showed that accounting for all the effects that could be partially confounded with a genetic additive effect is necessary for accurate EBV predictions, thus one hypothesis was that adding microbiota information in the model would allow a better estimation of the EBV’s prediction accuracies. This will need to be confirmed with additional datasets, but it would highlight a favorable impact of adding microbiota information in models for genetic selection.

#### High potential of gut microbiota to improve prediction accuracy of digestive efficiency traits

In the literature, Verschuren et al. [4] found a prediction accuracy of 0.43 for DC of organic matter. The sample size in the study of Verschuren et al. [4] was small (160 pigs), which may explain why the prediction accuracy was lower in their study than in ours. Likely, the standardized farming conditions in our project had a favorable impact on the prediction performance. For DE traits, the contribution of the gut microbiota to the variability of these traits has already been reported to be moderate to high, i.e. 58% for organic matter in the study of Verschuren et al. [4], and between 44 and 66% in a previous study that we performed with the same dataset [5]. Following this work, the results of the current study confirm that gut microbiota composition is promising for predicting these traits. From a biological point of view, the percentage of nutrients absorbed by the intestinal barrier depends largely on the fermentation activity carried out by the microorganisms hosted in the digestive tract [34, 35]. Since the composition of the gut microbiota is key to digestion, it is consistent with prediction accuracies for DE traits being improved when gut microbiota information is added in the model.

Several studies have shown that the gut microbiota composition changes throughout the life of an individual and that it is preferable to collect feces samples after post-weaning because gut microbiota composition becomes more stable with time [16, 35, 37]. The only study that has examined the best sampling time for trait prediction is that of Maltecca et al. [36] who reported no definitive result after 15 weeks of age and no data on DE. Thus, further studies are required to determine the appropriate age for collecting feces to best predict DE traits.

In the context of predicting phenotypes related to pig production, it has been observed that, compared to genomic information, the gut microbiota composition analyzed at 16 weeks of age provides valuable information for better prediction of DE traits. This suggests that incorporating gut microbiota information into the model, particularly in cases where there may be a confounding effect between genetic and microbiota influences, can lead to more accurate EBV. Previous studies by David and Ricard [33] have already indicated the potential benefits of including gut microbiota information for improving the accuracy of genetic selection for pigs. Therefore, the inclusion of gut microbiota composition in the model has the potential to positively impact the genetic selection process in pigs.

### Impact of diet on prediction accuracy values

Analysis of the prediction accuracies obtained in our study showed that they were generally consistent across the same diet, the opposite diet, and the combination of both diets and that there was only one significant difference between two scenarios for a specific trait, SBE connection, and within one model.

Initially, we hypothesized that prediction accuracies would be lower for opposite diets compared to within diets, particularly within the Micro model. This assumption was based on previous findings that indicated that the gut microbiota composition of the pigs in our study is influenced by the diet [12] as also reported for pigs from other French populations [38]. However, our initial hypothesis was not validated, as the prediction accuracies were similar both within and across diets. This could be attributed to limitations in our prediction scenarios. Although 1082 phenotypes were available for analysis, the cross-validation approach that we used required dividing the individuals into different subgroups, which resulted in a reduced number of individuals in the reference populations.

Moreover, for the prediction scenarios involving opposite diets, we expected lower prediction accuracies since only the most abundant OTU (2399 out of the initially detected 14,366) were retained for constructing the microbiota relationship matrix. However, we anticipated that rare OTU that are present in only one of the diets would contribute to altering the microbiota relationship values between individuals and potentially improve trait prediction. Nevertheless, this expectation was not confirmed in our study.

In summary, microbiota information proved to be a reliable predictor of DE traits, and the impact of the diet on prediction accuracies was limited.

### Impact of breeding environment on prediction accuracies

In this project, we found that prediction accuracies were significantly higher for FE and DE traits (0.27 and 0.47, respectively) when the reference and validation populations were connected by a shared breeding environment (SBE), compared to when they were not (0.16 and 0.40, respectively). It was anticipated that the prediction accuracies would be higher in the Micro model when the reference and validation populations shared a common SBE. This expectation was based on previous literature demonstrating that the breeding environment influences the composition of the intestinal gut microbiota [9, 11]. When pigs share a common environment, it is more likely that they will have similar gut microbiota compositions. Our results support this hypothesis, as we observed higher prediction accuracies in the Micro model when the reference and validation populations had a common breeding environment. However, we also observed higher prediction accuracies in the Gen model when the reference and validation populations shared a common SBE. This confirms that the genetic relatedness between the reference and validation populations also impacts prediction accuracies. Due to our experimental design, we cannot definitively determine whether the common breeding environment or a strong genetic connection is responsible for the improved prediction accuracy. Nevertheless, our findings indicate that the prediction accuracies for the FE and DE traits were enhanced when there was either an environmental or genetic connection between the reference and validation populations. It is worth noting that previous studies, such as the work of Habier et al. [39] and Legarra et al. [40], have already highlighted the benefits of including genetically related animals in the reference population to improve the precision of genomic predictions.

In summary, based on our dataset, we cannot conclusively determine whether it is the environmental or the genetic connection that improves prediction accuracies. However, our study provides valuable insights into the importance of having a certain connection between the reference and validation populations to enhance the prediction accuracies for growth, FE, and DE traits when incorporating microbiota or genomic information into the model.

## Conclusions

In conclusion, gut microbiota analysis is a promising approach to improve prediction accuracy of DE traits in growing pigs, which could be applied to pigs that are fed a range of diets from CO to alternative diets containing more dietary fibers. The prediction accuracies for these traits were significantly higher when microbiota information was added than when genomic information was added in the model. In contrast, including microbiota or genomic information in the model provided similar phenotypic prediction accuracies for FE traits. Further analyses on larger datasets and more diverse diets should be carried out to complement and verify these results. Finally, adding both microbiota and genomic information together in the model did not significantly increase the prediction accuracy of the different traits compared to adding either one of these two information. These results suggest a possible microbiota x genomic interaction, as recently highlighted in the literature, and should be verified in future studies.

### Supplementary Information

**Additional file 1: Figure S1.** Distribution of the percentage of individuals shared between the four different reference (in the upper part) and validation (in the bottom part) populations created when reference and validation populations were connected (in red) and not connected (in blue) by batches, under a conventional (CO in the middle) and a high-fiber (HF, to the right) diet, and when both diets were combined (CO+HF, to the left).

**Additional file 2: Table S1.** Number of shared sires between the reference and validation populations: impact of scenarios (COval/COref, HFval/HFref, COval/HFref, HFval/COref and COHFval/COHFref), and sire-breeding environment (SBE) connectivity across populations for each of the four samplings. **Table S2.** Detailed values of the mean prediction accuracy for digestive and feed efficiency traits presented in Figs. 2a, b, depending on the model (Gen, Micro and Micro+Gen), the scenario (COval/COref, HFval/HFref, COval/HFref, HFval/COref and COHFval/COHFref) and the sires and breeding environment (SBE) connection between reference and validation population, with the minimum and maximum values obtained among the four samples.

## Data Availability

The data supporting the findings of this study are available on request from the corresponding author. The data are not publicly available due to privacy or ethical restrictions. The sequences obtained for the microbiota are available and submitted to the Short-Read Archive with accession number PRJNA741111 (https://www.ncbi.nlm.nih.gov/sra/PRJNA741111).

## References

[1] Patience JF, Rossoni-Serão MC, Gutiérrez NA (2015). A review of feed efficiency in swine: biology and application. J Anim Sci Biotechnol.

[2] Déru V, Bouquet A, Labussière E, Ganier P, Blanchet B, Carillier-Jacquin C (2021). Genetics of digestive efficiency in growing pigs fed a conventional or a high-fibre diet. J Anim Breed Genet.

[3] Déru V, Bouquet A, Zemb O, Blanchet B, De Almeida ML, Cauquil L (2022). Genetic relationships between efficiency traits and gut microbiota traits in growing pigs being fed with a conventional or a high-fiber diet. J Anim Sci.

[4] Verschuren LMG, Schokker D, Bergsma R, Jansman AJM, Molist F, Calus MPL (2020). Prediction of nutrient digestibility in grower-finisher pigs based on faecal microbiota composition. J Anim Breed Genet.

[5] Déru V, Tiezzi F, Carillier-Jacquin C, Blanchet B, Cauquil L, Zemb O (2022). Gut microbiota and host genetics contribute to the phenotypic variation of digestive and feed efficiency traits in growing pigs fed a conventional and a high fiber diet. Genet Sel Evol.

[6] Camarinha-Silva A, Maushammer M, Wellmann R, Vital M, Preuss S, Bennewitz J (2017). Host genome influence on gut microbial composition and microbial prediction of complex traits in pigs. Genetics.

[7] Aliakbari A, Zemb O, Cauquil L, Barilly C, Billon Y, Gilbert H (2022). Microbiability and microbiome-wide association analyses of feed efficiency and performance traits in pigs. Genet Sel Evol.

[8] Velasco-Galilea M, Piles M, Ramayo-Caldas Y, Sánchez JP (2021). The value of gut microbiota to predict feed efficiency and growth of rabbits under different feeding regimes. Sci Rep.

[9] Mulder IE, Schmidt B, Stokes CR, Lewis M, Bailey M, Aminov RI (2009). Environmentally-acquired bacteria influence microbial diversity and natural innate immune responses at gut surfaces. BMC Biol.

[10] Zhang J. Development of gut microbiota in pigs and the effect of diet, antibiotics and other environmental factors. PhD thesis, Wageningen University. 2014.

[11] Le Sciellour M, Zemb O, Hochu I, Riquet J, Gilbert H, Giorgi M (2019). Effect of chronic and acute heat challenges on fecal microbiota composition, production, and thermoregulation traits in growing pigs. J Anim Sci.

[12] Déru V, Bouquet A, Zemb O, Blanchet B, Carillier-Jacquin C, Gilbert H. Influence d’une alimentation avec une teneur accrue en fibres sur le microbiote intestinal du porc en croissance. In: Proceedings of the 53rd Journées de la Recherche Porcine: 1–4 February 2021, Paris. 2021.

[13] Déru V, Bouquet A, Hassenfratz C, Blanchet B, Carillier-Jacquin C, Gilbert H (2020). Impact of a high-fibre diet on genetic parameters of production traits in growing pigs. Animal.

[14] R Core Team. R: a language and environment for statistical computing. Vienna: R Foundation for Statistical Computing; 2016.

[15] Labussière E, Ganier P, Conde JA, Janvier E, Van Milgen JJ. Development of a NIRS method to assess the digestive ability in growing pigs. In: Proceedings of the 70th annual meeting of the European Federation of Animal Science (EAAP): 26–30 August 2019, Gand. 2019.

[16] Zhao W, Wang Y, Liu S, Huang J, Zhai Z, He C (2015). The dynamic distribution of porcine microbiota across different ages and gastrointestinal tract segments. PLoS One..

[17] Callahan BJ, McMurdie PJ, Rosen MJ, Han AW, Johnson AJA, Holmes SP (2016). DADA2: high-resolution sample inference from Illumina amplicon data. Nat Methods.

[18] Quast C, Pruesse E, Yilmaz P, Gerken J, Schweer T, Yarza P (2013). The SILVA ribosomal RNA gene database project: improved data processing and web-based tools. Nucleic Acids Res.

[19] McMurdie PJ, Holmes S (2013). phyloseq: an R package for reproducible interactive analysis and graphics of microbiome census data. PLoS One..

[20] Purcell S, Neale B, Todd-Brown K, Thomas L, Ferreira MAR, Bender D (2007). PLINK: a tool set for whole-genome association and population-based linkage analyses. Am J Hum Genet.

[21] Ross EM, Moate PJ, Marett L, Cocks BG, Hayes BJ (2013). Investigating the effect of two methane-mitigating diets on the rumen microbiome using massively parallel sequencing. J Dairy Sci.

[22] VanRaden PM (2008). Efficient methods to compute genomic predictions. J Dairy Sci.

[23] Amadeu RR, Cellon C, Olmstead JW, Garcia AAF, Resende MFR, Muñoz PR (2016). AGHmatrix: R package to construct relationship matrices for autotetraploid and diploid species: a blueberry example. Plant Genome.

[24] Bates D, Mächler M, Bolker B, Walker S (2015). Fitting linear mixed-effects models using lme4. J Stat Soft.

[25] Kuznetsova A, Brockhoff PB, Christensen RHB (2017). lmerTest package: tests in linear mixed effects models. J Stat Soft.

[26] Pérez P, de los Campos G (2014). Genome-wide regression and prediction with the BGLR statistical package. Genetics.

[27] Weishaar R, Wellmann R, Camarinha-Silva A, Rodehutscord M, Bennewitz J (2020). Selecting the hologenome to breed for an improved feed efficiency in pigs—a novel selection index. J Anim Breed Genet.

[28] Tiezzi F, Fix J, Schwab C, Shull C, Maltecca C (2021). Gut microbiome mediates host genomic effects on phenotypes: a case study with fat deposition in pigs. Comput Struct Biotechnol J.

[29] Khanal P, Maltecca C, Schwab C, Fix J, Tiezzi F (2021). Microbiability of meat quality and carcass composition traits in swine. J Anim Breed Genet.

[30] Christensen OF, Börner V, Varona L, Legarra A (2021). Genetic evaluation including intermediate omics features. Genetics.

[31] Lu D, Tiezzi F, Schillebeeckx C, McNulty NP, Schwab C, Shull C (2018). Host contributes to longitudinal diversity of fecal microbiota in swine selected for lean growth. Microbiome.

[32] Verschuren LMG, Calus MPL, Jansman AJM, Bergsma R, Knol EF, Gilbert H (2018). Fecal microbial composition associated with variation in feed efficiency in pigs depends on diet and sex. J Anim Sci.

[33] David I, Ricard A (2019). A unified model for inclusive inheritance in livestock species. Genetics.

[34] Crespo-Piazuelo D, Estellé J, Revilla M, Criado-Mesas L, Ramayo-Caldas Y, Óvilo C (2018). Characterization of bacterial microbiota compositions along the intestinal tract in pigs and their interactions and functions. Sci Rep.

[35] Quan J, Cai G, Ye J, Yang M, Ding R, Wang X (2018). A global comparison of the microbiome compositions of three gut locations in commercial pigs with extreme feed conversion ratios. Sci Rep.

[36] Maltecca C, Lu D, Schillebeeckx C, McNulty NP, Schwab C, Shull C (2019). Predicting growth and carcass traits in swine using microbiome data and machine learning algorithms. Sci Rep.

[37] Pérez-Enciso M, Zingaretti LM, Ramayo-Caldas Y, de los Campos G (2021). Opportunities and limits of combining microbiome and genome data for complex trait prediction. Genet Sel Evol.

[38] Le Sciellour M, Labussière E, Zemb O, Renaudeau D (2018). Effect of dietary fiber content on nutrient digestibility and fecal microbiota composition in growing-finishing pigs. PLoS One..

[39] Habier D, Fernando RL, Dekkers JCM (2007). The impact of genetic relationship information on genome-assisted breeding values. Genetics.

[40] Legarra A, Robert-Granié C, Manfredi E, Elsen J-M (2008). Performance of genomic selection in mice. Genetics.

