# Supplementary figures and images for: The potential of microbiota information to better predict efficiency traits in growing pigs fed a conventional and a high-fiber diet
Source: Genet Sel Evol. 2024 Jan 19;56:8. doi: 10.1186/s12711-023-00865-4 (PMC10797989; doi:10.1186/s12711-023-00865-4)

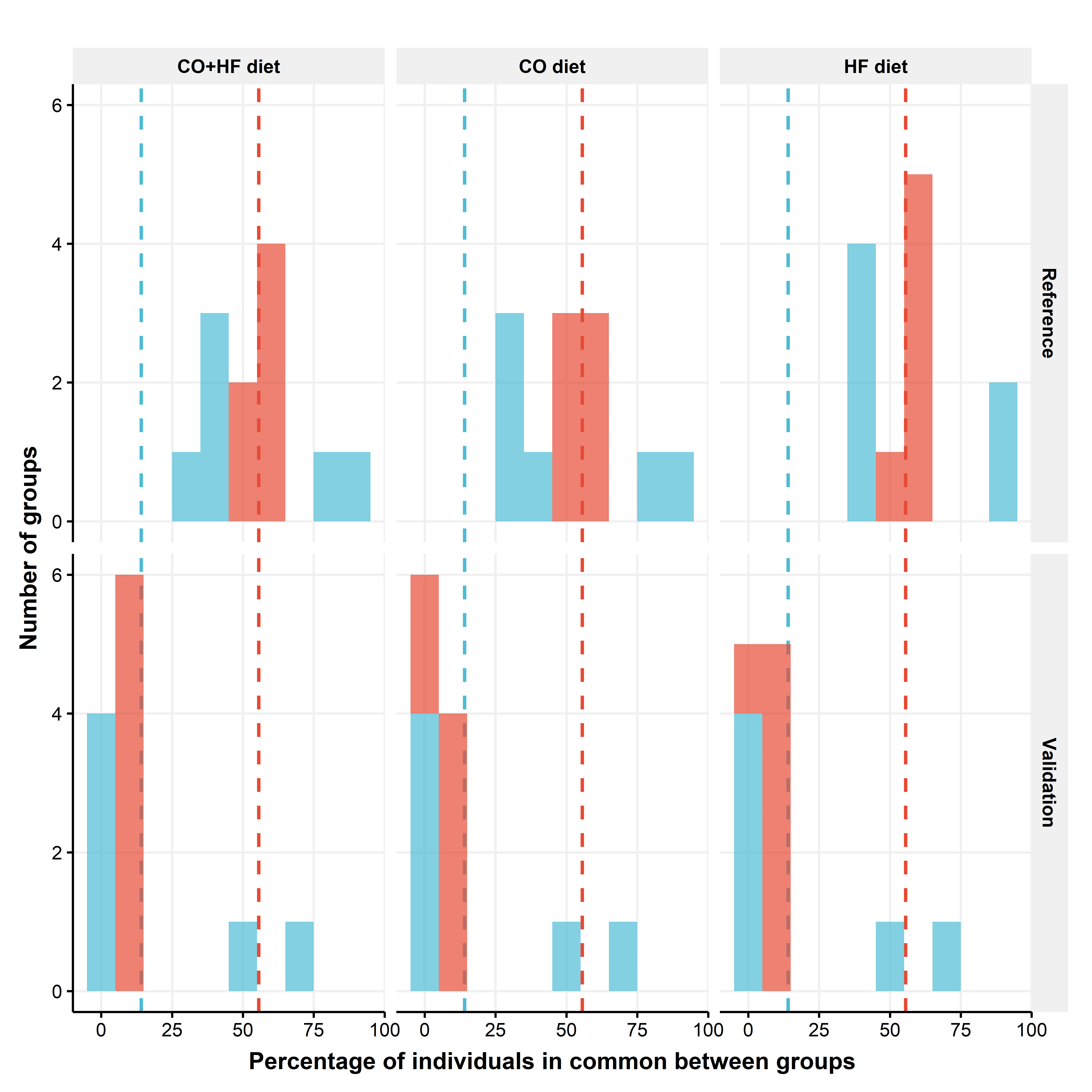

Supplement: Supplementary file 1 — Additional file 1: Figure S1. Distribution of the percentage of individuals shared between the four different reference (in the upper part) and validation (in the bottom part) populations created when reference and validation populations were connected (in red) and not connected (in blue) by batches, under a conventional (CO in the middle) and a high-fiber (HF, to the right) diet, and when both diets were combined (CO+HF, to the left). [file 12711_2023_865_MOESM1_ESM.png]
