# Supplementary material for: The potential of microbiota information to better predict efficiency traits in growing pigs fed a conventional and a high-fiber diet
Source: Genet Sel Evol. 2024 Jan 19;56:8. doi: 10.1186/s12711-023-00865-4 (PMC10797989; doi:10.1186/s12711-023-00865-4)
Supplement: Supplementary file 2 — Additional file 2: Table S1. Number of shared sires between the reference and validation populations: impact of scenarios (COval/COref, HFval/HFref, COval/HFref, HFval/COref and COHFval/COHFref), and sire-breeding environment (SBE) connectivity across populations for each of the four samplings. Table S2. Detailed values of the mean prediction accuracy for digestive and feed efficiency traits presented in Figs. 2a, b, depending on the model (Gen, Micro and Micro+Gen), the scenario (COval/COref, HFval/HFref, COval/HFref, HFval/COref and COHFval/COHFref) and the sires and breeding environment (SBE) connection between reference and validation population, with the minimum and maximum values obtained among the four samples. [file 12711_2023_865_MOESM2_ESM.docx]

**Additional file 2 Table S1**

| Scenario | SBE connection between ref. and val. | Number of sires in common between ref. and val. | | | |
| --- | --- | --- | --- | --- | --- |
|  |  | Sampling 1 | Sampling 2 | Sampling 3 | Sampling 4 |
| COref/COval | Connected | 33 | 35 | 32 | 28 |
|  | Not connected | 3 | 1 | 1 | 2 |
| HFref/HFval | Connected | 36 | 29 | 31 | 28 |
|  | Not connected | 1 | 1 | 3 | 2 |
| COref/HFval | Connected | 35 | 31 | 32 | 35 |
|  | Not connected | 1 | 1 | 2 | 2 |
| HFref/COval | Connected | 33 | 34 | 31 | 32 |
|  | Not connected | 3 | 1 | 2 | 1 |
| COHFref/COHFval | Connected | 59 | 56 | 58 | 57 |
|  | Not connected | 3 | 2 | 3 | 3 |

**Additional file 2 Table S2**

| **Scenarios** | **Trait** | **Model Gen** | |  | **Model Micro** | |  | **Model Micro+Gen** | |
| --- | --- | --- | --- | --- | --- | --- | --- | --- | --- |
|  |  | **Connected** | **Not Connected** |  | **Connected** | **Not Connected** |  | **Connected** | **Not Connected** |
| COHFref/COHFval | DCE | 0.07  [0.00 ;0.17] | 0.20  [0.13 ;0.36] |  | 0.59  [0.47 ;0.74] | 0.63  [0.49 ;0.73] |  | 0.57  [0.47 ;0.72] | 0.63  [0.51 ;0.73] |
| COHFref/COHFval | DCN | 0.20  [0.00 ;0.41] | 0.21  [0.09 ;0.42] |  | 0.65  [0.58 ;0.81] | 0.69  [0.56 ;0.82] |  | 0.66  [0.57 ;0.81] | 0.70  [0.56 ;0.82] |
| COHFref/COHFval | DCOM | 0.05  [-0.06 ;0.14] | 0.20  [0.13 ;0.39] |  | 0.59  [0.51 ;0.75] | 0.62  [0.48 ;0.72] |  | 0.57  [0.50 ;0.74] | 0.63  [0.50 ;0.71] |
| COHFref/COHFval | ADG | 0.23  [0.14 ;0.40] | 0.01  [-0.27 ;0.29] |  | 0.17  [0.07;0.34] | 0.16  [0.02 ;0.26] |  | 0.27  [0.14;0.50] | 0.04  [-0.13 ;0.31] |
| COHFref/COHFval | DFI | 0.14  [0.09 ;0.21] | 0.06  [0.02 ;0.11] |  | 0.33  [0.29 ;0.40] | 0.26  [0.12 ;0.41] |  | 0.22  [0.15 ;0.30] | 0.20  [0.14;0.32] |
| COHFref/COHFval | FCR | 0.20  [0.18 ;0.27] | 0.09  [0.02 ;0.17] |  | 0.09  [0.00;0.29] | 0.17  [0.04 ;0.29] |  | 0.24  [0.16;0.42] | 0.15  [0.04 ;0.21] |
| COHFref/COHFval | RFI | 0.28  [0.15 ;0.45] | 0.18  [0.12;0.26] |  | 0.24  [0.10 ;0.33] | 0.19  [0.15;0.27] |  | 0.33  [0.35 ;0.50] | 0.23  [0.17;0.26] |
| COref/COval | DCE | 0.07  [-0.12 ;0.27] | 0.27  [0.17 ;36] |  | 0.55  [0.44 ;0.62] | 0.54  [0.37 ;0.69] |  | 0.55  [0.44 ;0.65] | 0.56  [0.40 ;0.71] |
| COref/COval | DCN | 0.22  [-0.05 ;0.39] | 0.30  [0.26 ;0.34] |  | 0.69  [0.63 ;0.74] | 0.61  [0.50 ;0.73] |  | 0.70  [0.67 ;0.74] | 0.63  [0.52 ;0.73] |
| COref/COval | DCOM | 0.12  [-0.06 ;0.27] | 0.26  [0.19 ;0.29] |  | 0.55  [0.44 ;0.65] | 0.54  [0.37 ;0.72] |  | 0.56  [0.45;0.67] | 0.56  [0.40;0.72] |
| COref/COval | ADG | 0.02  [-0.17 ;0.08] | 0.24  [0.09 ;0.45] |  | 0.13  [0.02;0.28] | 0.13  [0.02 ;0.28] |  | 0.07  [0.09 ;0.24] | 0.28  [0.17;0.51] |
| COref/COval | DFI | 0.24  [0.19;0.32] | 0.18  [0.03 ;0.32] |  | 0.32  [0.03 ;0.56] | 0.25  [0.23 ;0.27] |  | 0.27  [0.10;0.46] | 0.29  [0.16 ;0.37] |
| COref/COval | FCR | 0.23  [0.14 ;0.34] | 0.12  [-0.08 ;0.08] |  | 0.27  [0.12;0.38] | 0.22  [0.16;0.34] |  | 0.33  [0.17;0.49] | 0.20  [0.14;0.32] |
| COref/COval | RFI | 0.19  [0.02 ;0.36] | 0.10  [0.06 ;0.14] |  | 0.36  [0.18 ;0.46] | 0.23  [0.12;0.43] |  | 0.38  [0.19 ;0.54] | 0.25  [0.17;0.42] |
| HFref/HFval | DCE | 0.20  [0.11 ;0.39] | 0.02  [-0.04 ;0.11] |  | 0.60  [0.51 ;0.671] | 0.62  [0.51 ;0.77] |  | 0.60  [0.50 ;0.70] | 0.61  [0.48 ;0.76] |
| HFref/HFval | DCN | 0.19  [0.13 ;0.31] | -0.01  [-0.05;0.28] |  | 0.65  [0.57 ;0.71] | 0.61  [0.44 ;0.79] |  | 0.65  [0.57 ;0.73] | 0.61  [0.43 ;0.79] |
| HFref/HFval | DCOM | 0.18  [0.07 ;0.35] | 0.00  [-0.06. ;0.06] |  | 0.59  [0.51 ;0.70] | 0.61  [0.50 ;0.75] |  | 0.60  [0.50 ;0.70] | 0.59  [0.48;0.74] |
| HFref/HFval | ADG | 0.27  [0.14;0.38] | 0.20  [0.09 ;0.35] |  | 0.23  [0.11;0.36] | 0.12  [0.03 ;0.22] |  | 0.34  [0.23 ;0.48] | 0.20  [0.00;0.34] |
| HFref/HFval | DFI | 0.14  [0.01 ;0.26] | 0.03  [-0.05 ;0.11] |  | 0.36  [0.27 ;0.46] | 0.21  [0.07 ;0.39] |  | 0.26  [0.14;0.38] | 0.11  [-0.06 ;0.32] |
| HFref/HFval | FCR | 0.17  [0.05 ;0.33] | 0.12  [-0.14 ;0.26] |  | 0.07  [0.11;0.22] | 0.20  [0.02;0.33] |  | 0.21  [0.08;0.29] | 0.18  [0.14;0.36] |
| HFref/HFval | RFI | 0.17  [0.05 ;0.26] | 0.10  [-0.01;0.31] |  | 0.26  [0.04 ;0.43] | 0.28  [0.09;0.36] |  | 0.28  [0.09 ;0.43] | 0.22  [0.06;0.36] |
| COref/HFval | DCE | 0.13  [0.03 ;0.25] | 0.10  [-0.06 ;0.19] |  | 0.60  [0.44 ;0.68] | 0.55  [0.40 ;0.72] |  | 0.55  [0.34 ;0.67] | 0.53  [0.35 ;0.71] |
| COref/HFval | DCN | 0.17  [-0.12 ;0.29] | 0.15  [-0.03 ;0.32] |  | 0.65  [0.56 ;0.74] | 0.54  [0.28 ;0.77] |  | 0.63  [0.57 ;0.74] | 0.55  [0.30 ;0.79] |
| COref/HFval | DCOM | 0.15  [0.00 ;0.25] | 0.10  [-0.05 ;0.21] |  | 0.59  [0.51 ;0.75] | 0.62  [0.48 ;0.72] |  | 0.57  [0.37 ;0.68] | 0.54  [0.39 ;0.69] |
| COref/HFval | ADG | 0.32  [0.23 ;0.37] | 0.32  [0.20 ;0.43] |  | 0.14  [0.07;0.36] | 0.23  [0.11 ;0.40] |  | 0.37  [0.27;0.52] | 0.34  [0.19 ;0.47] |
| COref/HFval | DFI | 0.21  [0.09 ;0.45] | 0.08  [0.11 ;0.20] |  | 0.27  [0.14 ;0.31] | 0.23  [0.08 ;0.37] |  | 0.15  [0.01 ;0.34] | 0.09  [0.05;0.16] |
| COref/HFval | FCR | 0.10  [-0.01 ;0.26] | 0.21  [0.07 ;0.30] |  | 0.03  [-0.09;0.08] | 0.17  [0.06;0.27] |  | 0.09  [0.05;0.16] | 0.25  [0.16 ;0.30] |
| COref/HFval | RFI | 0.10  [-0.07 ;0.21] | 0.12  [0.14 ;0.22] |  | 0.16  [0.09 ;0.21] | 0.25  [0.08;0.38] |  | 0.18  [0.08;0.29] | 0.22  [0.11;0.36] |
| HFref/COval | DCE | 0.15  [-0.15 ;0.28] | 0.05  [-0.06 ;0.25] |  | 0.55  [0.38 ;0.65] | 0.53  [0.47 ;0.56] |  | 0.52  [0.44 ;0.59] | 0.52  [0.44 ;0.59] |
| HFref/COval | DCN | 0.20  [0.05 ;0.34] | 0.00  [-0.12 ;0.19] |  | 0.65  [0.62 ;0.69] | 0.58  [0.51 ;0.64] |  | 0.66  [0.63 ;0.70] | 0.57  [0.49 ;0.64] |
| HFref/COval | DCOM | 0.14  [-0.18 ;0.27] | 0.07  [-0.03 ;0.26] |  | 0.56  [0.40 ;0.66] | 0.55  [0.48 ;0.63] |  | 0.55  [0.43 ;0.66] | 0.54  [0.47;0.64] |
| HFref/COval | ADG | 0.33  [0.11 ;0.45] | 0.25  [0.12 ;0.38] |  | 0.23  [0.08;0.43] | 0.18  [0.03 ;0.26] |  | 0.36  [0.29;0.43] | 0.28  [0.07 ;0.43] |
| HFref/COval | DFI | 0.27  [0.13;0.57] | 0.18  [0.11 ;0.26] |  | 0.39  [0.30;0.52] | 0.21  [0.15 ;0.29] |  | 0.42  [0.27 ;0.57] | 0.22  [0.14;0.33] |
| HFref/COval | FCR | 0.36  [0.22 ;0.52] | 0.10  [0.03 ;0.17] |  | 0.20  [0.11;0.42] | 0.07  [0.06;0.21] |  | 0.39  [0.24;0.55] | 0.11  [0.03 ;0.19] |
| HFref/COval | RFI | 0.41  [0.34 ;0.52] | 0.20  [0.13 ;0.26] |  | 0.30  [0.19 ;0.46] | 0.16  [-0.01;0.26] |  | 0.47  [0.34;0.55] | 0.25  [0.18;0.36] |
